# The size-weight illusion comes along with improved weight discrimination

**DOI:** 10.1371/journal.pone.0236440

**Published:** 2020-07-24

**Authors:** Christian Wolf, Knut Drewing

**Affiliations:** 1 Allgemeine Psychologie, Westfälische Wilhelms-Universität, Münster, Germany; 2 Allgemeine Psychologie, Justus-Liebig Universität, Giessen, Germany; Johns Hopkins University, UNITED STATES

## Abstract

When people judge the weight of two objects of equal mass but different size, they perceive the smaller one as being heavier. Up to date, there is no consensus about the mechanisms which give rise to this size-weight illusion. We recently suggested a model that describes heaviness perception as a weighted average of two sensory heaviness estimates with correlated noise: one estimate derived from mass, the other one derived from density. The density estimate is first derived from mass and size, but at the final perceptual level, perceived heaviness is biased by an object’s density, not by its size. Here, we tested the models’ prediction that weight discrimination of equal-size objects is better in lifting conditions which are prone to the size-weight illusion as compared to conditions lacking (the essentially uninformative) size information. This is predicted because in these objects density covaries with mass, and according to the model density serves as an additional sensory cue. Participants performed a two-interval forced-choice weight discrimination task. We manipulated the quality of either haptic (Experiment 1) or visual (Experiment 2) size information and measured just-noticeable differences (JNDs). Both for the haptic and the visual illusion, JNDs were lower in lifting conditions in which size information was available. Thus, when heaviness perception can be influenced by an object’s density, it is more reliable. This discrimination benefit under conditions that provide the additional information that objects are of equal size is further support for the role of density and the integration of sensory estimates in the size-weight illusion.

## Introduction

Human perception of our environment does not match one to one with its physical description, and discrepancies between the two reveal how the brain processes perceptual information [1]. One very salient discrepancy is given in the size-weight illusion: When people lift two objects of identical mass but different size, they perceive the smaller one as being heavier [2–4]. This size-weight illusion can be observed when objects are lifted simultaneously or sequentially using the same hand [5]. Moreover, the illusion persists when you know that two objects are equal in mass [6]. Although this illusion was first described more than one century ago [7, 8], there is still no consensus on the processes that give rise to this illusion.

One line of research tried to address the illusion from a top-down perspective, highlighting the role of expectations [9–12]. When lifting differently-sized objects that are made of the same material, larger objects are expected to be heavier. However, when two differently-sized objects are equal in mass, this expectation is violated and the illusion could be described as a contrast between weight and violated expectation [13]. Evidence for this notion comes from a study that manipulated participants’ expectations while they repeatedly lifted the same objects [11]. In this experiment, a small, medium or large cube was shown to participants. Participants were blindfolded shortly before they lifted the object using a handle and the small and large cube were exchanged by the medium-size cube. Perceived heaviness was higher when participants thought they were lifting the small compared to the large cube, showing that expectations can bias heaviness perception. Diverging results using a similar experimental manipulation were reported by Masin and Crestoni [14].

Even if size expectations can induce a size-weight illusion, they cannot account for the full strength size-weight illusion with sensory size information present, because when induced by expectations only, the illusion magnitude is reduced to approximately one third [11]. It may be noteworthy that expectations also affect the forces that are used to lift an object: the smaller of two objects with identical masses is lifted with less force than its large counterpart [15]. However, with repeated lifting, forces are adapted to the real object mass while lifting the object, whereas the illusion persists [11, 13, 16–18]. Thus, the illusion cannot be explained by differences in the forces applied during lifting.

Another line of research tried to address the illusion from a bottom-up perspective. The first sensory explanation was provided by Charpentier [7] who reasoned that lifting objects with identical masses, but different sizes leads to differences in the skin contact area, and differences in mass distribution over the skin cause the illusion. However, the illusion also occurs when objects are lifted on a string (and thus have the same contact area) and their size is only perceived visually, even if illusion strength is somewhat reduced in this visual illusion compared to a size-weight illusion where participants can gain haptic information [4, 19, 20]. Also a size-weight illusion induced by echolocation has been reported [21], further emphasizing that the size-weight illusion is not restricted to peripheral processing or the haptic sense, but rather caused by a central mechanism [22]. Another argument for the sensory nature of the size-weight illusion is that the magnitude of the illusion depends on the quality with which size information can be perceived: The illusion magnitude decreases the less reliable size estimates are, no matter whether size information is obtained haptically or visually [20, 23]. Perceptual reliability is inversely proportional to the variance of a perceptual estimate [24, 25]. Thus, the more reliable a perceptual estimate, the less variable it is. Reliability can therefore be considered an index of perceptual precision and depends on the perceptual quality with which a stimulus is perceived. Differences in the quality of haptic size estimates might also explain why a larger illusion magnitude was reported for lifting with both hands compared to one-handed lifting [26].

Many researchers have emphasized a role for density in the size-weight illusion [20, 27–32], because two objects of equal mass but different size will consequently also differ in density, with the smaller of the two being denser than the other. We recently suggested a model [20] that describes perceived heaviness as a weighted combination of two heaviness estimates with correlated noise: one estimate derived from mass, the other one derived from density. The integration of mass and density estimate is supposed to follow the principles of maximum-likelihood integration [24, 25, 33]. Whereas mass can directly be perceived, density must first be derived from mass and size. However, at the final perceptual level, perceived heaviness is biased by an object’s density, not by its size. Because both estimates share the same information about mass, their noise will not be independent but correlated [33]. The model predicts that perceived heaviness increases with physical mass and with physical density, it predicts that the denser of two equally-weighted objects is perceived as heavier, that the perceived difference depends on the difference in physical density and it predicts that the illusion strength depends on the quality with which size and thus density information can be perceived. Empirical results supported these predictions [20].

The aim of the present work is to test a further model prediction: weight discrimination should be better in lifting conditions in which density information can be integrated to distinguish weight, as compared to conditions in which density information is not available. In particular, we tested whether weight discrimination of equal-size objects is better when size information on the objects is available (and thus a size-weight illusion can occur) as compared to conditions lacking size information. Note that size information is essentially uninformative by itself, because objects are of equal-size. However, the size information allows to estimate density. In the equal-size objects density covaries with mass and according to our model, heaviness estimates from density are integrated with estimates from mass. According to the principles of maximum-likelihood integration, the integration of different estimates increases perceptual reliability as compared to single-estimate situations, which is reflected in better discrimination performance. Improved discrimination performance is in turn considered strong evidence for sensory integration [24, 25].

We measured just-noticeable differences (JNDs) as an index of discriminability in a haptic and a visual weight discrimination experiment. Just-noticeable differences and discrimination performance are inversely related: A more reliable perceptual estimate and thus better weight discrimination is reflected in a lower JND value. In every trial, participants had to sequentially lift two equal-size objects, one standard stimulus and one of multiple comparisons (method of constant stimuli) and decide which of the two feels heavier (two-interval forced choice task, 2IFC). In a control condition common to both experiments, participants were blindfolded and lifted standard and comparisons on a string so that they had neither visual nor haptic information about size. When objects are lifted without visual or haptic cues about size or material, perceived heaviness can be described as a function of mass without any influence of other object characteristics [19, 20, 34]. Consequently, for both experiments we expected the highest JND values in this string/blindfold control condition.

**Fig 1:**
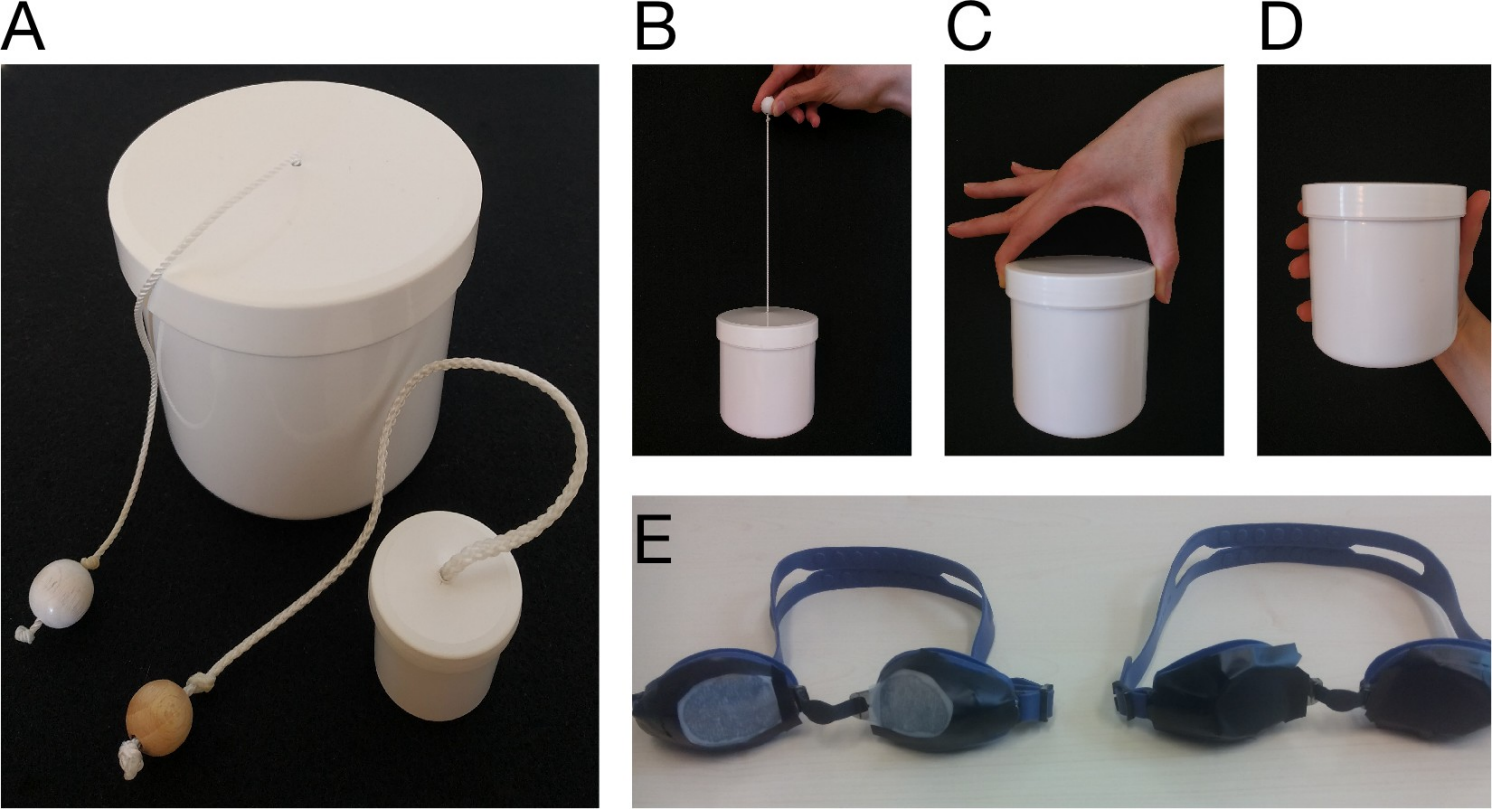
Stimuli and critical manipulations. (A) Exemplary stimuli of the large and the small set. For the visual experiment and the string condition in the haptic experiment, stimuli were equipped with a string and a wooden bead. Unlike depicted in the figure, all wooden beads used in the experiments were white. (B-D) Grip types used in the haptic experiment. Objects were lifted (B) on a string, (C) using a precision grip or (D) by fully enclosing the object. (E) Goggles used in the visual discrimination experiment to blindfold participants (left) and to impair vision (right).

In the haptic experiment (Experiment 1), participants additionally lifted the objects with a precision grip or by fully enclosing the object. These two grip types have been shown to give rise to an intermediate and a full-strength haptic size-weight illusion [20]. Moreover, fully enclosing the object with the hand is associated with better haptic size discrimination compared to a precision grip [20]. In the visual experiment (Experiment 2), participants always lifted objects on a string, and we manipulated whether or how well they could see the lifted object. In addition to the control condition, participants were either wearing vision-impairing goggles (Fig 1) or had full vision of the object. Again, these two conditions had been shown to give rise to an intermediate and a full-strength visual size-weight illusion [20]. We expect better weight discrimination (lower JNDs) in lifting conditions associated with a size-weight illusion. Specifically, we expect for the haptic experiment that JNDs are highest in the control condition, intermediate in the precision grip condition and lowest when participants can fully enclose the object. Likewise, we expect for the visual experiment that JNDs are highest when participants are blindfolded, intermediate when they wear vision-impairing goggles and lowest when they have full vision of the objects. Given that the illusion magnitude is higher for the haptic compared to the visual size-weight illusion [4, 19, 20], we expect the weight discrimination benefit to be more pronounced in the haptic experiment.

## Methods

### Participants

All participants were undergraduate or graduate students from Giessen University, had normal or corrected-to-normal vision and declared to have no severe neurological impairments, nor any sensory or motor deficits related to their hands or arms. Participants received 8€/h or course credit as compensation for participating. All participants provided written informed consent prior to testing. The experiments were approved by the local ethics committee of FB06 at Giessen University and were conducted in accordance with the declaration of Helsinki (2013). For the haptic experiment, we recorded data from 10 individuals (mean age: 25 years, age range: 20–32, 5 female, 1 left-handed according to self-report). For the visual experiment, we recorded data from 24 participants (6 male) with a mean age of M = 25 years (SD = 5, range = 18–38, 1 left-handed according to self-report). To reduce the load on individual participants of the visual experiment, we chose to reduce the number of observations for every psychometric function and thus for every individual. As a consequence of this and of the lower expected effect size in the visual experiment, we chose to record a higher number of participants for the visual experiment.

### Stimuli

Stimuli of both experiments were white cylindrical plastic cans with a screw cap. See [20] for a more detailed description and image of stimuli. Cans were filled with a mixture of iron or tungsten powder together with either silicone or polyurethane foam.

#### Haptic experiment (Experiment 1)

For the haptic experiment, stimuli either belonged to a small volume set (V_small_ = 32 cm^3^) or a large volume set (V_large_ = 596 cm^3^). Within each set, masses of stimuli ranged in 9 steps from 180 to 220 g (180, 186, 194, 198, 200, 202, 206, 214 and 220 g) with 200 g being the standard stimulus and the remaining 8 being comparisons. In the string condition we attached a string of 20 cm length to each screw cap. At the end of the string, there was a wooden bead with a diameter of 13 mm. Due to string and bead, actual masses were slightly higher than noted above. Deviances were around 2 g for every stimulus. However, these differences were small and did not affect the mass difference between stimuli. To keep stimulus names consistent across conditions and experiments, we will address stimuli with the masses they have without string and bead.

#### Visual experiment (Experiment 2)

In the visual experiment, we used stimuli of the small set equipped with string and bead. Compared to Experiment 1, we discarded the 198 g and 202 g stimuli and added comparison stimuli of 170, 200 and 230 g. Thus, we had comparison stimuli of 170, 180, 186, 194, 200, 206, 214, 220 and 230 g and a standard stimulus of 200 g. For all conditions, we attached a string of 20 cm length to each screw cap. To blindfold participants, we used diving goggles equipped with a non-transparent black foil so that participants could not see anything (Fig 1E). To impair vision, we used the same type of diving goggles, but equipped them with a transparent colorless foil (“d-c-fix 7”). This pair of goggles blurred vision and only coarse patterns and shapes could be identified. At the beginning of every condition, participants were given enough time to adjust the strap of each goggle so that it felt comfortable.

### Design and procedure haptic experiment

The design of the haptic experiment comprised two within-participants variables, stimulus set (small set, large set) and grip type (string, precision grip, enclosure). In every trial of the haptic experiment, participants had to indicate which of two objects felt heavier, the standard stimulus or a comparison (method of constant stimuli). For every participant we fitted 6 psychometric functions, one for every condition. From every function, we derived the just-noticeable difference (JND, see data analysis) to assess discrimination performance.

At the beginning of each trial, the experimenter placed the first stimulus on the felt pad in front of the blindfolded participant and led his/her dominant hand towards it. Participants had to lift each stimulus and weigh it up and down twice before placing it down again on the felt pad. In the string condition, participants were instructed to extend their dominant hand at about 30 cm above the table with palm down and the experimenter placed the stimulus’ wooden bead between the participants’ thumb and index fingers. In the precision grip condition participants had their hand above the felt pad like in the string condition, but were asked to pre-shape their hand to a precision grip. The experimenter then moved the hand towards the object so that the thumb touched the screw cap and participants were asked to move the index finger towards the screw cap without moving the thumb to another location or by touching the object with another finger or parts of the hand. In the enclosure condition, participants had their arm resting on the table and the experimenter moved their hand towards the object so the thumb touched the object. Participants were allowed to move the thumb to any other location and asked to have as much skin contact as possible when lifting the object. After placing down the first stimulus, the experimenter exchanged both stimuli and the second stimulus had to be lifted in the same manner. Participants responded after having placed the second stimulus on the felt pad. Across blocks, the order of comparison and standard stimulus was balanced for every comparison stimulus. In every block all 8 comparisons were lifted once in a random order. There have been 20 blocks for every combination of grip type and set. Thus, the whole experiment consisted of 960 trials per participant (8 comparisons × 20 blocks/repetitions × 2 sets × 3 grip types) and lasted 5–7 hours, depending on the individual pace. The experiment was split in sessions of up to two hours with regular breaks. Participants were told at the beginning of the experiment that they could take a break at any time. Each session contained a manifold of 10 blocks and the number of sessions was not consistent across participants. All participants completed 10 blocks of the string condition for both sets (half of them started with the small, the other with the large set) before completing 10 blocks in the precision grip and the enclosure condition. This order was repeated for every participant in the second half of the experiment. A fixed order of conditions was chosen to prevent a transfer of information into conditions providing less haptic information.

### Design and procedure visual experiment

In the visual discrimination experiment all objects were lifted on a string and we manipulated the quality of visual information. The design thus comprised the within-participant factor visibility: Participants were either blindfolded, had full vision of the lifted objects or their vision was impaired by wearing diving goggles that were equipped with a foil.

Compared to the haptic experiment we only recorded data for one of the two stimuli sets to reduce the burden on each participant. We chose to use stimuli from the small set. In our previous study stimuli of the large set were perceived as lighter due to the size-weight illusion [20]. Consequently, better weight discrimination for these large objects could not only be due to density integration, but potentially also because of the reduced perceived intensity: According to Weber’s law discrimination thresholds increase (or decrease) with an increasing (or decreasing) stimulus intensity. To ensure that our results are not caused by changes in perceived intensity, we used stimuli of the small set. These objects were reported as heavier due to the size-weight illusion [20]. Thus, when using the small set any potential effect of perceived intensity would have counteracted the benefit due to density integration.

For the visual experiment, setup and lifting procedure were identical to the string condition in the haptic experiment. There have been 18 repetitions for every comparison in every of the three visibility conditions, resulting in (9 comparisons x 18 repetitions) 162 trials per condition and thus per psychometric function. Overall, the whole experiment consisted of (162 trials x 3 visibility conditions) 486 trials and lasted between 3.5 and 4.5 hours split into two sessions with regular breaks. Comparing the second and first part of the haptic experiment revealed no evidence for a transfer of information about density. We therefore chose a fully balanced design for the visual experiment to additionally assure that the data are not influenced by potential order effects. Thus, the order of visibility conditions was fully balanced across participants such that every of the six possible orders of the three conditions was recorded equally often. In the second session, the order of conditions was reversed for every individual: For example, if a participant successively completed 9 blocks in the impaired vision, full vision and blindfolded condition in the first session, he/she would start the second session with the blindfolded condition before proceeding to the full vision and impaired vision condition.

As a manipulation check, we measured visual acuity with the impairing diving goggles (impaired vision) and without any goggles (full vision) after the weight discrimination experiment. The order of conditions was balanced across participants. To measure visual acuity, we used a printed version of Landolt rings that was laid down at the felt pad, thus at the location where the stimulus was placed during the discrimination experiment. Landolt rings had a gap at one of eight possible locations with gap sizes corresponding to one fifth of their diameter. Gap sizes and thus also ring diameter varied in 15 steps with gap sizes of 3, 2, 1.6, 1.4, 1.2, 1, 0.8, 0.6, 0.4, 0.3, 0.2, 0.1, 0.06, 0.04 and 0.03 cm. For every gap size there were four different rings. Every participant started with the largest gap size. If three or more gap directions had been indicated correctly, the participants continued with the next smaller gap size. We defined visual acuity thresholds as the minimum gap size that could be reliably discriminated. If participants were able to discriminate all gap sizes correctly, we used the smallest gap size (0.03 cm) as threshold.

### Data analysis

For every comparison in every condition, we computed the proportion it was judged heavier than the standard. We then fitted a cumulative Gaussian to the data using psignifit 4 [35] in MATLAB R2018b (The Mathworks, Natick, MA, USA). Just-noticeable differences (JNDs) were defined as standard deviation of the fitted cumulative Gaussian. The remaining parameters of the psychometric function were allowed to vary during the fitting procedure, but restricted within a generous and reasonable range of values: Points of subjective equalities (mean of Gaussian) were restricted to values between 150 and 250 g and guess and lapse rates (lower and upper asymptotes of the Gaussian) to values below 0.225. Inferential statistics were performed in JASP (Version 0.10.2; [36]). For ANOVAs we report partial eta squared $\eta_{p}^{2}$ as an estimate of effect size. For *t*-tests, we report Cohen’s *d*.

## Results

### Haptic experiment

**Fig 2:**
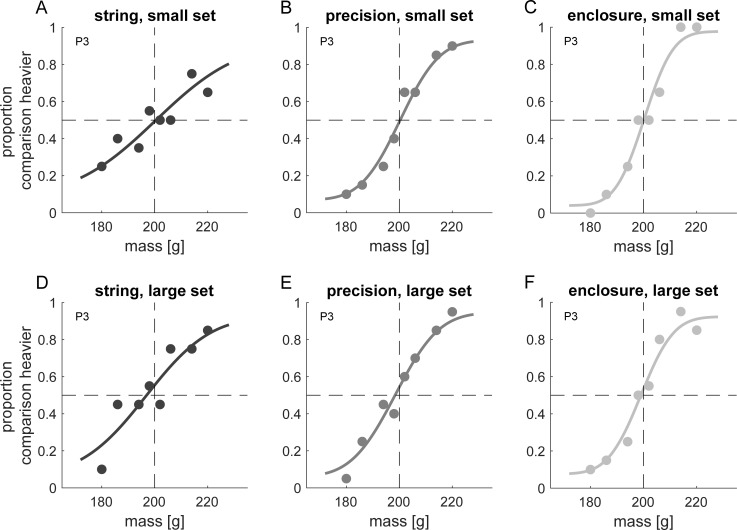
Psychometric functions of one representative participant (P3) for the haptic experiment. Panels show psychometric functions (cumulative Gaussians) together with proportion of responses that each of the eight comparisons was judged heavier than the 200 g standard. Panels in the upper row refer to the small set of stimuli, panels in the lower row to the large set. Every column shows data from a different lifting condition (A, D: string; B, E: precision grip; C, F: enclosure). Just-noticeable differences (JNDs) are the standard deviation of the fitted Gaussian.

**Fig 3:**
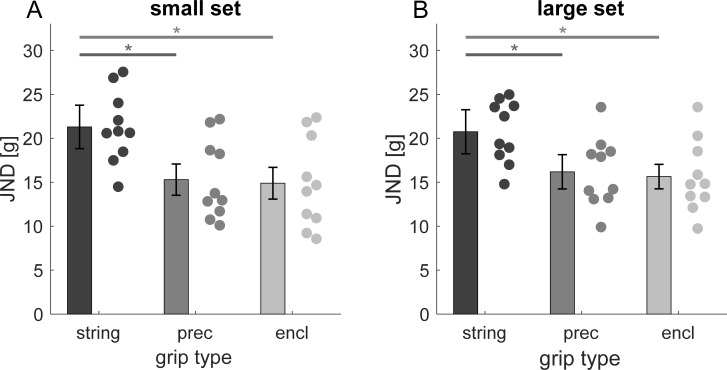
Aggregated JND values in the haptic experiment for the small (A) and the large stimulus set (B). Bars represent the average value with error bars being 95% confidence-intervals of within-participant variability according to Cosineau [37]. Data points are results of individuals (prec: precision grip; encl: enclosure). Lines and asterisks denote a significant difference between two conditions.

Fig 2 shows psychometric functions for one participant in the three lifting conditions for each of the two sets. Average JND values (Fig 3) are highest in the string condition and lower in conditions that give rise to a size-weight illusion (precision grip, enclosure). We compared JND values using a 3 x 2 (grip type x set size) ANOVA. The ANOVA revealed a main effect of grip type, *F*(2,18) = 12.348, *p* < 0.001, $\eta_{p}^{2}$ = 0.578, but neither a main effect of set, *F*(1,9) = 0.139, *p* = 0.718, $\eta_{p}^{2}$ = 0.015, nor a set × grip type interaction could be observed, *F*(2,18) = 0.387, *p* = 0.684, $\eta_{p}^{2}$ = 0.041. Compared to the string condition JND values of both sets were reduced in the precision grip, small set: *t*(9) = 3.63, *p* = 0.005, *d* = 1.148, large set: *t*(9) = 3.30, *p* = 0.009, *d* = 1.043, and enclosure condition, small set: *t*(9) = 3.30, *p* = 0.009, *d* = 1.043, large set: *t*(9) = 2.814, *p* = 0.02, *d* = 0.89,. We observed no difference in JND values between the precision grip and enclosure condition, small set: *t*(9) = 0.382, *p* = 0.712, *d* = 0.121, large set: *t*(9) = 0.384, *p* = 0.71, *d* = 0.121.

### Visual experiment

We first checked whether our manipulation of visibility was successful: Without goggles 22 out of 24 participants could reliably discriminate the smallest gap size of Landolt rings (0.03 cm, maximum: 0.06 cm). When wearing goggles, all participants required a larger ring and gap size in order to discriminate the location of the gap (median: 0.8 cm, range: 0.3 cm– 1.6 cm), *t*(23) = 13.37, *p* < 0.001, *d* = 2.73, indicating that wearing the foil-equipped goggles successfully impaired vision.

**Fig 4:**
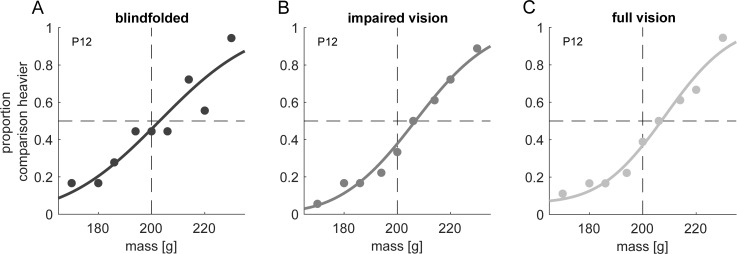
Psychometric functions of one representative participant (P12) for the visual experiment. Plots are equivalent to Fig 2.

**Fig 5:**
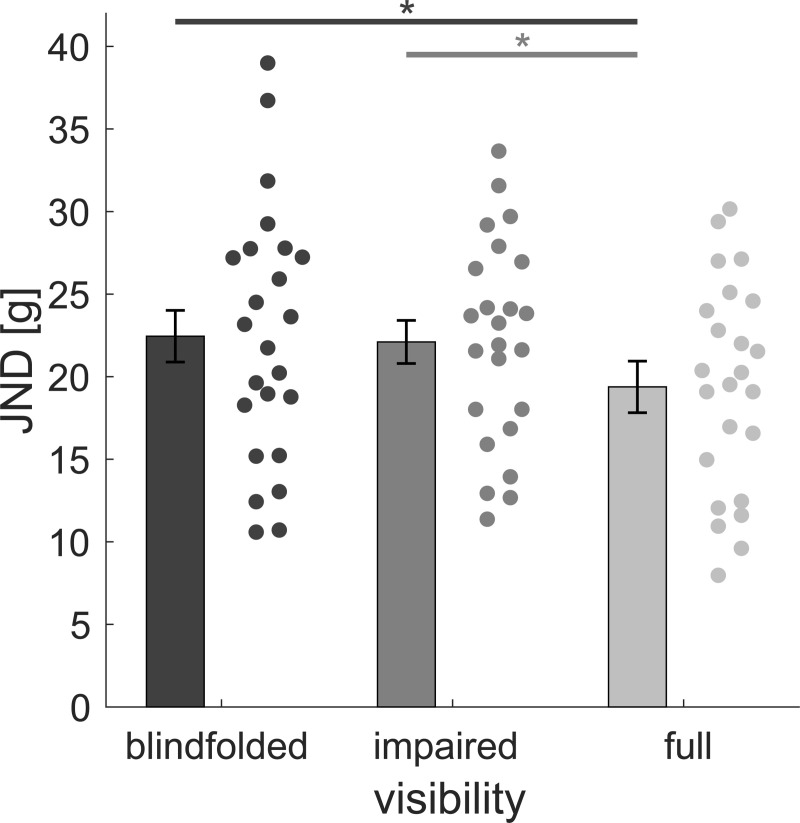
Aggregated JND values in the visual experiment for the blindfolded (blind), impaired vision (poor) and full vision (full) condition. Bars represent the average with 95% confidence-intervals of within-participant variability according to Cosineau [37]. Data points are results of individuals. Lines and asterisks denote a significant difference between two conditions.

Fig 4 shows psychometric functions of one representative participant for the three visibility conditions. Average JND values (Fig 5) were highest in the blindfolded and lowest in the full vision condition. A one-way repeated-measures ANOVA revealed a significant main effect of visibility, *F*(2, 46) = 3.288, *p* = 0.046, $\eta_{p}^{2}$ = 0.125. JND values in the full vision condition were significantly reduced compared to the blindfolded condition, *t*(23) = 2.115, *p* = 0.045, *d* = 0.432, and the impaired vision condition, *t*(23) = 2.209, *p* = 0.037, *d* = 0.451. There was no reduction in JND values in the impaired vision compared to the blindfolded condition, *t*(23) = 0.28, *p* = 0.782, *d* = 0.057.

## Discussion

In two experiments we measured weight discrimination performance (by just-noticeable differences) of equal-size objects in conditions shown to be associated with either no, an intermediate or a full-strength size-weight illusion. In a first experiment, we manipulated the access to and quality of haptic size information while participants were blindfolded, and visual size information was thus not available. In a second experiment, we manipulated the access to and quality of visual size information while participants lifted objects on a string, and haptic size information was not available. Just-noticeable differences were reduced in conditions associated with a full-strength illusion compared to conditions associated with no illusion. This was true for both volume sets tested in the haptic experiment as well as for the one set tested in the visual experiment. This suggests that a full-strength size-weight illusion coincides with better weight discrimination. As we did not measure the size-weight illusion during the experiment, this relationship can therefore be considered an indirect one. However, given that the illusion is highly robust and does not even disappear when participants are told that objects are equal in mass [16], and given that stimuli and perceptual conditions in each single trial of the present experiments were almost identical to corresponding conditions of previous experiments, we have reason to believe that the lifting conditions used in the present experiments did not only give rise to a size-weight illusion in our previous study [20] but also in the present experiments. Yet, including a measurement of the illusion could have revealed whether and how individual illusion strength and benefit in terms of discrimination performance are related.

For the intermediate conditions, precision grip and impaired vision [20], the pattern of results was less clear. In the haptic experiment, JNDs of both sets in the precision grip condition were lower as compared to the no-illusion condition, but not higher than in the full-strength enclosure condition. In the visual experiment, JNDs were not lower in the impaired vision as compared to the no-illusion condition, but higher as compared to the full-strength visual illusion condition. This dichotomous pattern of results compared to the predicted gradual pattern might either reflect a lack of statistical power or it could reflect the presence of an additional process. The results of both intermediate conditions would be grossly consistent with an interpretation in terms of lack of power, particularly if one considers the previously reported illusion magnitude in these conditions [20]. The magnitude of the size-weight illusion in our previous study was captured by a model parameter (density weight) that expresses how much the perceptual judgment was affected by density information. Larger density weights (up to 1) indicate a stronger illusion and a weight of 0 indicates the absence of an illusion. For the haptic experiment of our previous study [20], the density weight in the precision grip condition (0.31) was closer to the full haptic illusion (0.42) compared to the absence of an illusion, whereas in the corresponding visual experiment, the density weight in the impaired visual condition (0.14) was closer to the absence of an illusion compared to the full strength visual illusion (0.29). In addition to a lack of statistical power, one may argue that in the haptic experiment, a performance benefit could also have arisen due to differences in the way objects are lifted rather than due to the availability of size information: differences in grip type might have caused changes in the forces applied during lifting which might in turn have caused differences in sensitivity to weight [38–41]. Moreover, weight sensitivity might have been directly affected by the different grip types, either because of the different skin contact areas or because of the direct contact with the object compared to lifting it with a tool (string) as tool use has been shown to alter aspects of perception [42, 43]. In addition to density integration, these aspects might have contributed to the JND differences between the string condition and the two conditions providing direct haptic contact with the object. However, such objections to our interpretation are not true for the visual discrimination experiment where grip type and thus also the skin contact area and presumably also the forces applied during lifting were the same across conditions. The same is true for possible order effects: Whereas all participants of the haptic experiment started with the string condition, the order of conditions was fully balanced across participants of the visual experiment. The appearance of the stimuli was not directly helpful in assessing their weight, because their appearance was identical. Taken together, we take our results as support for our hypothesis that lifting conditions associated with a full-strength size-weight illusion coincide with better weight discrimination due to the integration of density information, which provides a parsimonious explanation for both visual and haptic findings.

The finding of lower JNDs in conditions that give rise to the size-weight illusion had been predicted from our mass-density model on heaviness perception [20]. The model predicted that weight discrimination of equal-size objects is better when size information on the objects is available (and thus a size-weight illusion can occur) as compared to conditions lacking size information. This is because, in the equal-size objects density covaries with mass and according to the model heaviness estimates from density are integrated with estimates from mass. The reliability, i.e. the inverse of variance, is supposed to be higher when the mass estimate and the density estimate are integrated, and people are prone to the illusion. In turn, a higher reliability should be reflected in better weight discrimination and thus lower JNDs. According to maximum-likelihood integration, this improved reliability is strong evidence for integration of information arising from multiple senses [24, 25] or from different cues within a sensory modality [44]. This benefit in terms of reliability is also predicted when the two integrated estimates are not independent, but when their noise is correlated. However, in this case, the improvement in reliability is diminished [20, 33].

Earlier findings suggested that there might be an optimal density for weight discrimination [30]. This dates back to the idea of “non-illusory” weights first mentioned by Huang [45] and experimentally introduced by Ross [34]: The perceived weight of objects of different sizes is measured under two lifting conditions, once with and once without any cues to size. For every apparent material, the size (and thus density) where perceived weight with and without size cues match was considered the “non-illusory” weight [34]. Using this method, Ross [34] found density values of 1.7 g/cm^3^ for tin stimuli, 0.14 g/cm^3^ for polystyrene stimuli to be non-illusory. In a subsequent study Ross and Gregory [30] measured weight discrimination for different apparent materials and densities. In their weight discrimination experiments, participants always had access to size information. These non-illusory weights were found to have the optimal density that was associated with better weight discrimination and any deviation from this density was associated with poorer discrimination performance resulting in a U-shaped relationship [30]. This optimal density hypothesis is in line with the idea of robust integration that states that information is only integrated when two signals are similar, but not when they are clearly conflicting [46, 47]. However, the optimal density hypothesis is not in line with our present results. If there was an optimal density associated with better weight discrimination, then we would have expected a different pattern of results for the two sets used in the haptic experiment. Specifically, we would have expected different JND values for both sets in conditions associated with a size-weight illusion. However, this was not the case. This very similar pattern for both sets would only be consistent with the optimal density hypothesis when densities of both sets were on symmetrical positions of the U-shaped relationship reported by Ross and Gregory [30]. In this case an even stronger performance benefit would be expected for an intermediate density.

Overall, this leaves us with an odd situation. When objects are lifted without any information to size, then perception is not prone to illusory influences and physical and apparent weight correlate almost perfectly [19, 20, 34]. However, here we show that in these conditions without size information, weight discrimination is less reliable compared to conditions which are prone to the size-weight illusion. Thus, it seems that the perceptual system sacrifices perceptual consistency for the sake of better, more reliable discrimination. This would be helpful for a system that has evolved to distinguish between different materials of differing weight rather than to accurately predict the weight of objects.
